# Limy bile (Milk of calcium bile) associated with gall stones discovered incidentally during laparoscopic cholecystectomy

**DOI:** 10.1016/j.ijscr.2019.07.032

**Published:** 2019-07-22

**Authors:** Sardar Hassan Arif, Ayad Ahmad Mohammed

**Affiliations:** Department of Surgery, College of Medicine, University of Duhok, Duhok City, Iraq

**Keywords:** Limy bile, Milk of calcium bile, Gallstones, Cholethiasis, Laparoscopic cholecystectomy

## Abstract

**Introduction:**

Limy bile is very rare condition in which the gall bladder is filled with a whitish material formed mainly of calcium carbonate. It may be associated with gall stones and present with epigastric and right upper quadrant pain, fever, nausea, and jaundice.

The diagnosis is easily made during radiology showing opacification of the gallbladder or the bile ducts on plain abdominal X-rays and CT-scan. Most patients are treated with cholecystectomy.

**Case presentation:**

A 35-year-old lady was complaining from right hypochondrial pain for 8 months. The pain was mainly at the late night and relieved with analgesics. During examination the patient had no jaundice and during abdominal examination there was deep tenderness in the right hypochonrdium. Ultrasound of the gallbladder showed evidence of multiple gall stones with bile sludge. During laparoscopic cholecystectomy the gall bladder was found to be filled with white creamy substance and multiple gall stones. The patient discharged next day with no postoperative complications.

**Conclusion:**

Limy bile is a rare clinical entity of unknown etiology but obstruction of the cystic duct may be the most important step in its occurrence. The condition needs cholecystectomy as in most of the cases it is associated with gall stones. There are no reported long term sequelae and long term follows up is not recommended.

## Highlights

• Limy bile referred to high level of calcium carbonate inside the bile causing the characteristic whitish appearance .
• The condition may be asymptomatic and may be associated with gall stone disease.
• It is readily diagnosed by radiology showing the gall bladder or sometimes the biliary system are filled with a radiopaque material.

## 1 Introduction

Limy bile or sometimes called milk of calcium bile is very rare condition in which the gall bladder or in some rare occasions the biliary canaliculi are filled with a thick, whitish, and paste like material that varies in consistency from liquid form to a semisolid material which is mainly formed form calcium carbonate.When both the gall bladder and the biliary tree are filled with this limy bile it is called double localization [1,2].

This condition was reported for the first time in 1911 by Churchman, after that this clinical entity drew the attention of the physicians and more cases have been reported. With respect to the term lime-milk bile which was used by Volkmann in 1929, this term is not always applicable when the contents are solid and the term concretions may be more suitable. The condition is more common in females and mostly affects individuals aged above 40 years [2,3].

Regardless whether this condition is associated with gall stones or not, the main clinical symptoms are epigastric and right upper quadrant pain, fever, nausea, jaundice. Examination of the patients may show jaundice, tenderness in the right hypochonrdium and when there is obstruction of the neck of the gall bladder there may be palpable gall bladder [4].

The diagnosis of this condition may be easily made during radiology which appears as opacification of the gallbladder or the bile ducts, this is may be evident on plain abdominal X-rays, CT-scan or MRI examinations of the abdomen. [2,5]

The work of this case report has been reported in line with the SCARE criteria. [6]

## 2 Patient information

A 35-year-old lady was complaining from right hypochondrial pain and attacks of nausea for 8 months. The pain was mainly at the late night awaking the patient from sleep and relieved with analgesics. The past medical and the surgical history were nor relevant.

### 2.1 Clinical findings

During general examination the patient had no jaundice with normal vital signs, and during abdominal examination there was deep tenderness in the right hypochonrdium with no palpable masses and no organ enlargement.

### 2.2 Diagnostic assessment

**Fig. 1:**
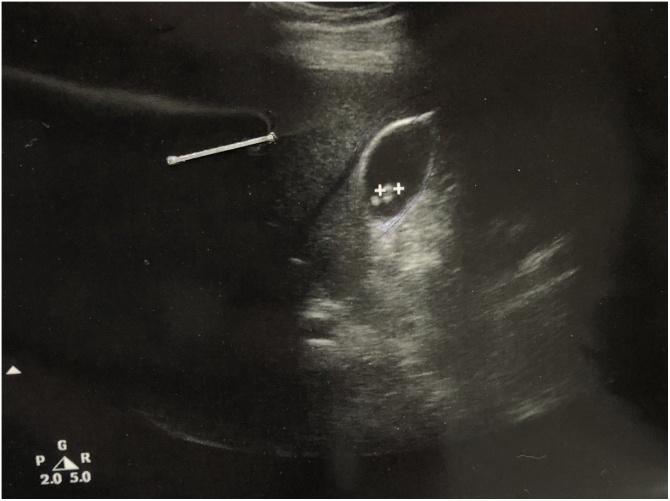
An ultrasound picture of the gall bladder showing the presence of gall stones with thick bile and slight increase in the wall thickness.

Ultrasound of the gallbladder showed evidence of multiple gall stones with bile sludge, with normal caliber of the common bile duct and other organ examination showed no significant abnormalities. Fig. 1.

## 3 Therapeutic intervention

**Fig. 2:**
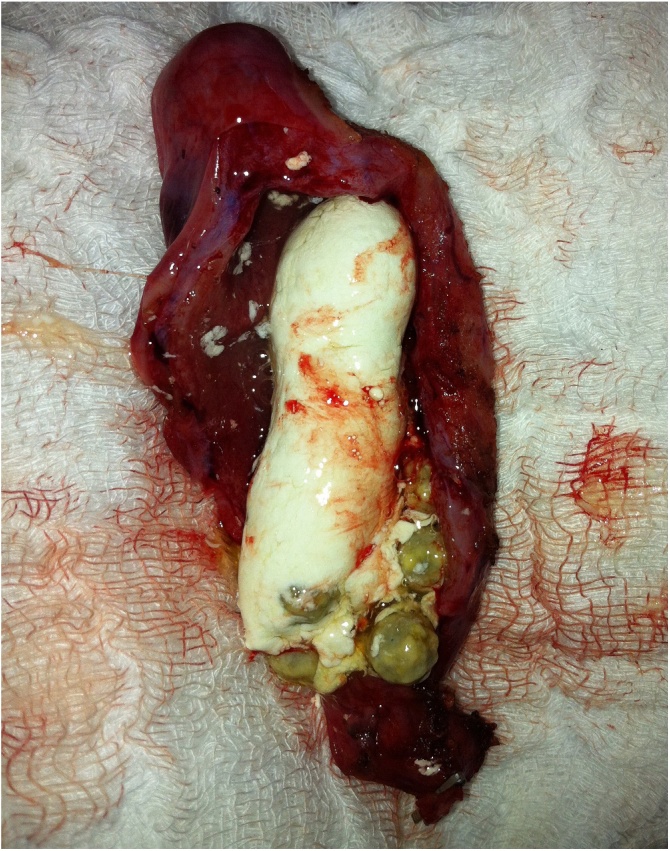
Showing the gall bladder cavity filled with the whitish creamy limy bile with multiple gall stones inside.

During laparoscopic cholecystectomy and after extraction of the gall bladder from the abdomen, the gall bladder found to be filled with white creamy substance and multiple gall stones and the condition of limy bile was considered. Fig. 2.

### 3.1 Follow-up and outcomes

The patient stayed in the surgical ward for one day and discharged next day with no postoperative complications.

## 4 Discussion

The formation of the limy bile can be explained by many theories; one of them is blockage of the cystic duct is an essential factor for the formation of limy bile and gall stones are present in the majority of the patients, the other possible cause is that affected patients have been found to have abnormal systemic calcium metabolism, or it may be due to low bile pH, the normal hepatic bile pH is above 8, the pH of the bile inside the gallbladder is ranging between 6.6-9. It may also be due to abnormal calcium molecules that are formed in the gall-bladder. Small amount of calcium is absorbed from the gallbladder during bile concentration. Calcium may form bile salt complexes which are insoluble, and in the presence of diseased gall-bladders such soluble complexes are then precipitated [7].

This condition may be associated with some complications such as cholecystitis whether acute or chronic, pancreatitis, or obstructive jaundice [1].

Some cases are treated conservatively when it is not associated with gall stones. Spontaneous disappearance of this condition had been reported, when the condition is associated with gall stones such cases are treated with cholecystectomy which can be done laparoscopically as the gall stone disease is a common complaint and laparoscopic cholecystectomy is one of the most commonly performed operations worldwide [1,2,8].

When the stones are present in the common bile duct the stones may be extracted using the endoscopic retrograde cholangiopancreatography or in cases of very large stones the patient may need operative exploration of the common bile duct and extraction of the stone [4].

The association of cancer of the gallbladder or cholangiocarcinoma is not clear and no enough data are available but most authors agree that the coexistence of cancer with limy bile is easily missed on imaging and they encourage the operative management once this condition is diagnosed [4].

## Conflicts of interest

The author has no conflicts of interest to declare.

## Sources of funding

None.

## Ethical approval

Ethical approval has been exempted by my institution for reporting this case.

## Consent

Written informed consent was obtained from the patient for publication of this case report and accompanying images.

## Author contribution

Dr Ayad Ahmad Mohammed and Dr Sardar Hassan Arif are the surgeons who performed the operation.

Dr Ayad Ahmad Mohammed and Dr Sardar Hassan Arif. contributed to the concept of reporting the case and the patient data recording.

Drafting the work, design, and revision done by Dr Ayad Ahmad Mohammed.

Dr Ayad Ahmad Mohammed took the consent from the patient for publishing the case.

Final approval of the work to be published was done by Dr Ayad Ahmad and Dr Sardar Hassan Arif.

## Registration of research studies

This work is case report and there is no need of registration

## Guarantor

Dr Ayad Ahmad Mohammed is guarantor for the work

## Provenance and peer review

Not commissioned.
